# Small bowel GIST harboring concurrent KIT exon 9 duplication and SDHC mutation: A case report

**DOI:** 10.18632/oncotarget.28863

**Published:** 2026-05-04

**Authors:** Cameron B. Speyer, Kyle D. Klingbeil, Manando Nakasaki, Sarah M. Dry, Arun S. Singh, Karo K. Arzoo, Fritz C. Eilber, Joseph G. Crompton

**Affiliations:** ^1^Department of Surgery, Division of Surgical Oncology, UCLA David Geffen School of Medicine, Los Angeles, CA 90095, USA; ^2^Jonsson Comprehensive Cancer Center, David Geffen School of Medicine, University of California Los Angeles, Los Angeles, CA 90095, USA; ^3^Department of Pathology, University of California Los Angeles, Los Angeles, CA 90095, USA; ^4^Department of Medicine, Division of Hematology-Oncology, University of California Los Angeles, Los Angeles, CA 90095, USA

**Keywords:** GIST, KIT duplication, SDHC mutation, genetic testing, case report

## Abstract

Gastrointestinal stromal tumors (GISTs) are molecularly defined by oncogenic alterations that predict clinical behavior and response to therapy. Activating mutations in KIT or PDGFRA characterize most GISTs and confer sensitivity to imatinib, whereas succinate dehydrogenase (SDH)–deficient GISTs lack these mutations and are typically imatinib resistant. These molecular subtypes are generally considered mutually exclusive. We report a rare case of a small bowel GIST harboring both a somatic KIT exon 9 A502_Y503 duplication and a germline inactivating SDHC mutation (p.R50C). The patient received neoadjuvant high-dose imatinib with a marked radiographic and metabolic response, followed by complete surgical resection. Pathology demonstrated spindle cell GIST with significant treatment effect and retained SDHB expression. This case suggests that oncogenic KIT signaling may remain the dominant driver of GIST behavior despite the presence of a germline SDHC mutation and highlights the importance of integrated molecular interpretation in GIST management.

## INTRODUCTION

Gastrointestinal stromal tumors (GISTs) are the most common mesenchymal tumors of the gastrointestinal tract and are thought to arise from, or share a common precursor with, the interstitial cells of Cajal [1, 2]. Advances in molecular characterization have revealed that GISTs represent a biologically diverse group of tumors with distinct oncogenic drivers, clinical behavior, and therapeutic vulnerabilities. In clinical practice, the molecular genotype of a GIST is the primary determinant of systemic treatment selection and predicted response [3].

Approximately 75–85% of GISTs harbor activating mutations in the receptor tyrosine kinases *KIT* or *PDGFRA*, leading to constitutive signaling and sensitivity to tyrosine kinase inhibitors such as imatinib [4, 5]. In contrast, GISTs lacking mutations in *KIT* or *PDGFRA*—historically termed wild-type GISTs—constitute a heterogeneous subgroup that includes tumors associated with neurofibromatosis type 1, *BRAF* mutations, gene fusions, and succinate dehydrogenase (SDH) deficiency [4, 5]. SDH-deficient GISTs are biologically distinct, often occurring in younger patients, frequently arising in the stomach, and characteristically resistant to imatinib [4, 5].

Importantly, activating *KIT/PDGFRA* mutations and SDH deficiency have traditionally been considered mutually exclusive mechanisms of GIST tumorigenesis. Here, we describe a rare case of a small bowel GIST harboring both a somatic *KIT* exon 9 duplication and a germline *SDHC* mutation. This unusual molecular profile presented diagnostic and therapeutic challenges and provided insight into the hierarchy of oncogenic drivers in GIST.

## CASE REPORT

A 68-year-old man with no significant past medical history presented to the emergency department with acute on chronic abdominal pain. The patient reported approximately two months of intermittent abdominal discomfort and ten days of progressive pain, bloating, and constipation. The patient identified as Caucasian, non-Hispanic, of European descent, reported no family history of malignancy or hereditary cancer syndromes, denied tobacco or alcohol use and had no prior colonoscopy.

Computed tomography (CT) of the abdomen and pelvis demonstrated a large, heterogeneous intraperitoneal mass measuring approximately 10 × 18 × 18 cm in the right lower quadrant. The mass was adherent to multiple loops of small bowel and abutted the sigmoid colon and urinary bladder, without evidence of bowel obstruction, mesenteric or retroperitoneal lymphadenopathy, or invasion of adjacent organs. Magnetic resonance imaging (MRI) confirmed a large mixed solid and cystic mass occupying a significant portion of the lower abdomen and pelvis.

**Figure 1:**
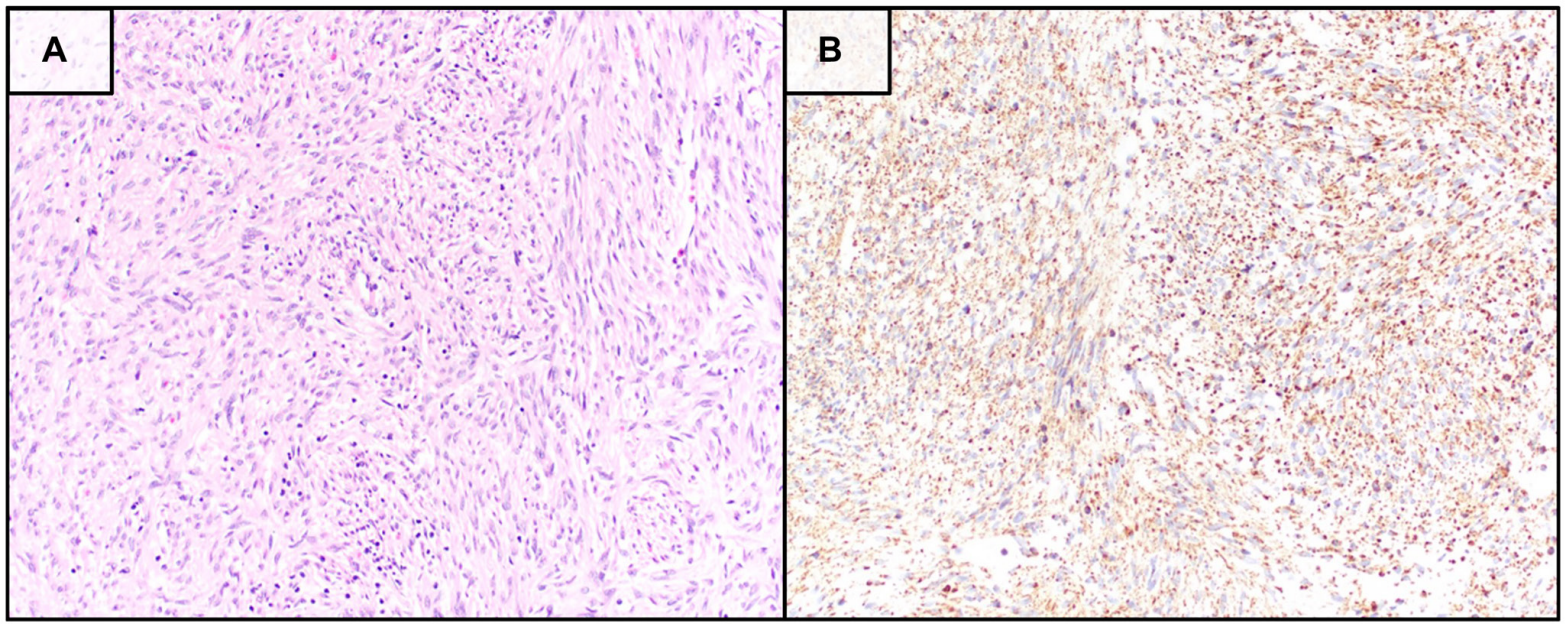
Histopathologic and immunohistochemical features of the tumor biopsy. (**A**) Hematoxylin and eosin–stained section (20× magnification) demonstrating a spindle cell neoplasm with morphology consistent with gastrointestinal stromal tumor (GIST). (**B**) Immunohistochemical staining for succinate dehydrogenase subunit B (SDHB) showing retained cytoplasmic expression within tumor cells, visualized with diaminobenzidine chromogen and hematoxylin counterstain (20× magnification). Retained SDHB expression suggests preservation of SDH complex integrity despite the presence of a germline SDHC variant.

Image-guided core needle biopsy revealed a spindle cell neoplasm consistent with a GIST (Figure 1A), with strong diffuse positivity for CD117 and DOG1, focal positivity for CD34 and smooth muscle actin, and negativity for S100. β-catenin staining demonstrated membranous positivity, and the Ki-67 proliferation index was <2% [6–8].

Next-generation sequencing of the tumor (NeoGenomics, NeoType^™^ Analysis, GIST and Soft Tissue Tumor Profile) identified an exon 9 *KIT* A502_Y503 duplication and an inactivating *SDHC* p.R50C mutation, with low tumor mutational burden, microsatellite stability, and negative PD-L1 expression. No alterations were detected in *PDGFRA, BRAF, KRAS, NRAS, HRAS, NF1,* or *SDHB*. The *SDHC* variant was present at an allele frequency of 49.4%. Subsequent germline testing (Natera^™^, Empower^™^ Comprehensive) confirmed a heterozygous SDHC c.148C>T (p.R50C) variant, and the patient was referred for genetic counseling.

**Figure 2:**
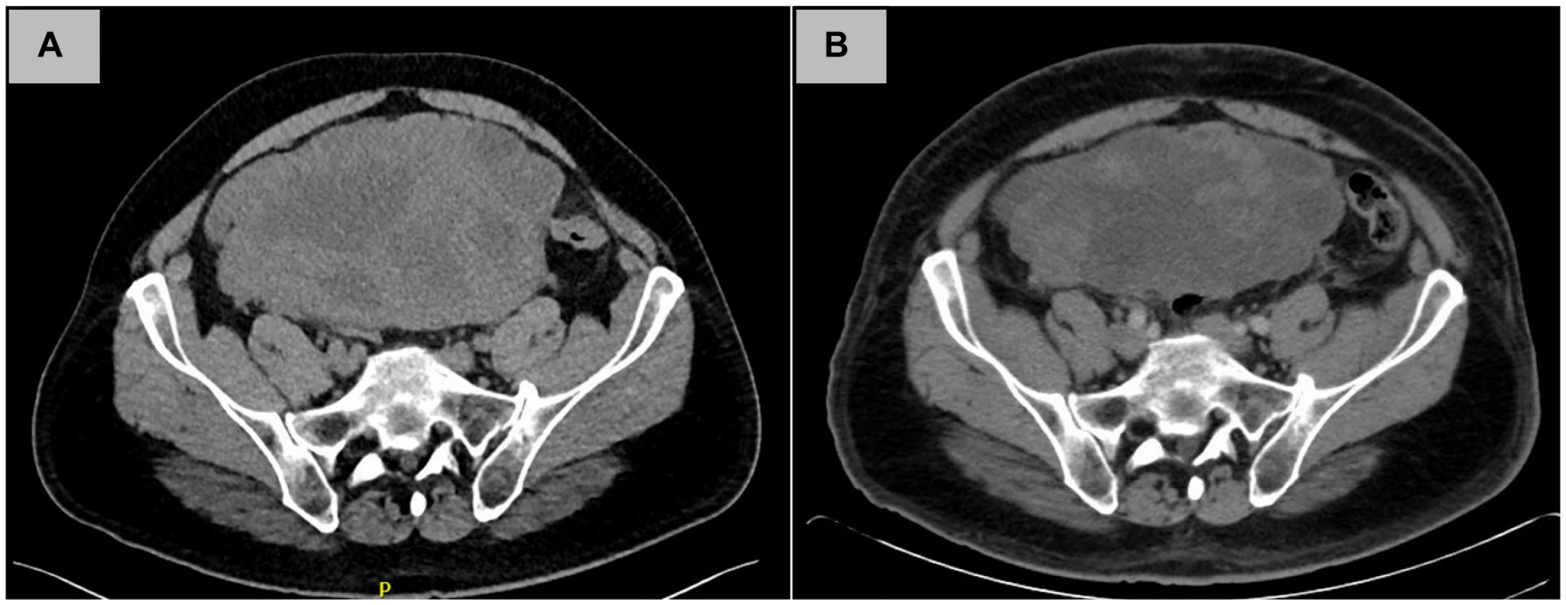
Radiographic tumor response to neoadjuvant imatinib therapy. (**A**) Contrast-enhanced computed tomography (CT) scan at initial presentation demonstrating a large heterogeneous mass occupying the lower abdomen and pelvis. (**B**) Repeat CT scan obtained after three months of imatinib therapy showing interval decrease in tumor size and internal complexity, consistent with partial radiographic response.

The patient’s case was discussed in a multidisciplinary tumor board. Neoadjuvant systemic therapy was recommended to facilitate resection and reduce surgical morbidity. In light of the *KIT* exon 9 duplication, imatinib therapy was initiated [9]. Additional staging was performed as germline *SDH* mutations are associated with paragangliomas, pheochromocytomas, renal cell carcinoma, and Carney–Stratakis syndrome [4, 10, 11]. CT imaging of the chest, abdomen, and pelvis showed no evidence of metastatic disease or associated neoplasms other than the ileal GIST (Figure 2A), and baseline positron emission tomography (PET)/CT demonstrated avid uptake within the mass epicenter (SUVmax 10.2).

The patient began imatinib at 400 mg daily, which was well tolerated, and the dose was escalated to 800 mg daily in accordance with guidelines for *KIT* exon 9-duplicated GIST [9]. Treatment-related toxicities were limited to mild anemia and peripheral edema. After three months of therapy, repeat imaging demonstrated a partial response, with reduction of the mass to 8 × 16 × 14 cm (Figure 2B) and a marked decrease in metabolic activity (SUVmax 2.9). The patient completed six months of neoadjuvant imatinib before proceeding to surgery.

**Figure 3:**
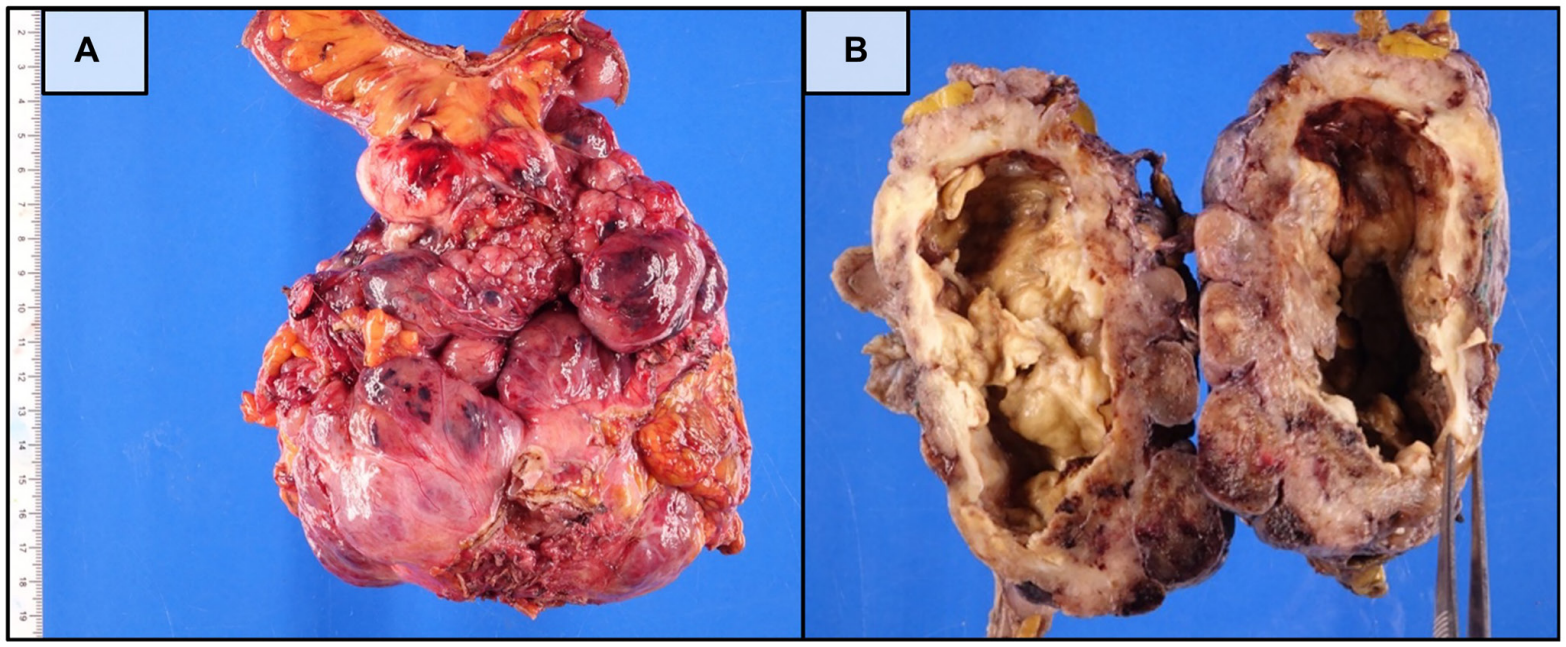
Gross pathologic features of the surgically resected small bowel GIST. (**A**) Fresh surgical specimen demonstrating a large, well-circumscribed mass arising from the small bowel. (**B**) Formalin-fixed specimen bisected to reveal extensive central necrosis and cavitation consistent with treatment effect following neoadjuvant imatinib therapy.

The patient then underwent successful surgical resection of the GIST. Exploratory laparotomy revealed a large necrotic mass arising from the ileum, adherent to the anterior abdominal wall, omentum, and dome of the bladder, without invasion of the sigmoid colon or rectum. En bloc resection was performed, including approximately 10-cm of small bowel, omentum, peritoneum, and a full-thickness portion of the bladder (Figure 3A, 3B). Final pathology demonstrated a 17-cm spindle cell GIST with 8 mitoses per 5 mm² and 50–60% tumor necrosis, consistent with partial treatment effect. All surgical margins were negative. Pathologic stage was Stage IIIB (ypT4N0M0). Postoperatively, the patient recovered uneventfully. The patient resumed high-dose imatinib as adjuvant therapy based on NCCN guidelines [12]. At five months following surgery, surveillance imaging showed no evidence of disease recurrence.

## DISCUSSION

This case illustrates an unusual molecular profile in GIST, combining an activating *KIT* exon 9 duplication with a germline inactivating *SDHC* mutation—alterations that are traditionally regarded as mutually exclusive. GISTs are molecularly stratified based on oncogenic drivers, and this stratification has direct therapeutic implications. Activating *KIT* or *PDGFRA* mutations predict sensitivity to imatinib, whereas SDH-deficient GISTs represent a distinct biologic entity that is typically resistant to standard tyrosine kinase inhibitors.

*KIT* exon 9 duplications account for a minority of KIT-mutant GISTs and are most commonly associated with tumors arising in the small intestine [2, 13–16]. These tumors exhibit a more aggressive clinical phenotype and demonstrate improved outcomes with higher-dose imatinib compared with standard dosing. In contrast, SDH-deficient GISTs usually arise in the stomach, occur in younger patients with a female predominance, and often present as multifocal disease [4, 5]. Many of these SDHC-deficient tumors are present as part of the Carney Triad, a nonheritable syndrome related to SDHC hypermethylation [4].

*SDHC*-mutated GIST, however, is not well described in the literature. Experimental studies have shown p.R50C affects SDHC function [17]. Germline *SDHC* mutations are associated with Carney-Stratakis syndrome, a familial syndrome characterized by paragangliomas and GIST [5]. Loss of SDHB expression by immunohistochemistry is a defining feature of SDH-deficient tumors and serves as a reliable diagnostic surrogate for SDH complex instability [18]. Paradoxically, the final surgical pathology demonstrated retained SDHB protein in this case (Figure 1B).

SDH complex dysfunction is thought to occur during tumorigenesis through accumulation of succinate, leading to pseudohypoxia and epigenetic dysregulation that silences an SDH-complex subunit via methylation [1, 4]. Loss of any SDH subunit destabilizes the entire complex [19]. However, development of SDH-deficient GIST generally requires biallelic inactivation of an SDH subunit gene [20]. In contrast, KIT-mutated GISTs arise from activating gain-of-function mutations in the KIT receptor tyrosine kinase, in which alteration of a single allele is sufficient to produce constitutive kinase signaling and drive tumorigenesis independent of SDH complex dysfunction [3]. In the present case, the identified germline loss-of-function SDHC mutation likely resulted in the inactivation of a single allele, which appears insufficient to destabilize the SDH complex, as evidenced by retained SDHB protein expression on immunohistochemistry. Therefore, the SDHC alteration is unlikely to have contributed significantly to tumorigenesis in this patient’s GIST.

Despite the presence of a germline *SDHC* mutation in our patient, several features strongly support KIT signaling as the dominant oncogenic driver. The tumor demonstrated a robust response to imatinib, a finding that would be unexpected in an SDH-deficient GIST. Localization to the small bowel, retained SDHB expression, and tumor response to high-dose imatinib are all characteristic of *KIT* exon 9–duplicated GIST rather than SDH-deficient GIST [21, 22].

This case underscores the importance of interpreting germline and somatic findings within their clinical and pathologic context. As broad next-generation sequencing becomes increasingly common, clinicians will encounter tumors with multiple potentially pathogenic alterations. Determining which mutation is the true oncogenic driver is critical for appropriate therapeutic decision-making. Given the inherent limitations of a single case report, these findings should be interpreted with caution, and additional studies are needed to determine the clinical and biological significance of concurrent KIT and SDHC alterations in GIST.

## CONCLUSIONS

We report a rare case of small bowel GIST harboring both a somatic *KIT* exon 9 duplication and a germline *SDHC* mutation. Despite the coexistence of these alterations, the tumor behaved in a manner consistent with KIT-driven disease and demonstrated a marked response to high-dose imatinib. This case highlights the complexity of GIST molecular biology and emphasizes the need for integrated molecular, pathologic, and clinical assessment to guide treatment.

## References

[1] Corless CL, Barnett CM, Heinrich MC. Gastrointestinal stromal tumours: origin and molecular oncology. Nat Rev Cancer. 2011; 11:865–78. 10.1038/nrc3143. 22089421

[2] Hirota S, Isozaki K, Moriyama Y, Hashimoto K, Nishida T, Ishiguro S, Kawano K, Hanada M, Kurata A, Takeda M, Muhammad Tunio G, Matsuzawa Y, Kanakura Y, et al. Gain-of-function mutations of c-kit in human gastrointestinal stromal tumors. Science. 1998; 279:577–80. 10.1126/science.279.5350.577. 9438854

[3] Blay JY, Kang YK, Nishida T, von Mehren M. Gastrointestinal stromal tumours. Nat Rev Dis Primers. 2021; 7:22. 10.1038/s41572-021-00254-5. 33737510

[4] Boikos SA, Pappo AS, Killian JK, LaQuaglia MP, Weldon CB, George S, Trent JC, von Mehren M, Wright JA, Schiffman JD, Raygada M, Pacak K, Meltzer PS, et al. Molecular Subtypes of KIT/PDGFRA Wild-Type Gastrointestinal Stromal Tumors: A Report From the National Institutes of Health Gastrointestinal Stromal Tumor Clinic. JAMA Oncol. 2016; 2:922–28. 10.1001/jamaoncol.2016.0256. 27011036 PMC5472100

[5] Janeway KA, Kim SY, Lodish M, Nosé V, Rustin P, Gaal J, Dahia PL, Liegl B, Ball ER, Raygada M, Lai AH, Kelly L, Hornick JL, and NIH Pediatric and Wild-Type GIST Clinic. Defects in succinate dehydrogenase in gastrointestinal stromal tumors lacking KIT and PDGFRA mutations. Proc Natl Acad Sci U S A. 2011; 108:314–18. 10.1073/pnas.1009199108. 21173220 PMC3017134

[6] Miettinen M, Makhlouf H, Sobin LH, Lasota J. Gastrointestinal stromal tumors of the jejunum and ileum: a clinicopathologic, immunohistochemical, and molecular genetic study of 906 cases before imatinib with long-term follow-up. Am J Surg Pathol. 2006; 30:477–89. 10.1097/00000478-200604000-00008. 16625094

[7] Miettinen M, Wang ZF, Lasota J. DOG1 antibody in the differential diagnosis of gastrointestinal stromal tumors: a study of 1840 cases. Am J Surg Pathol. 2009; 33:1401–8. 10.1097/PAS.0b013e3181a90e1a. 19606013

[8] West RB, Corless CL, Chen X, Rubin BP, Subramanian S, Montgomery K, Zhu S, Ball CA, Nielsen TO, Patel R, Goldblum JR, Brown PO, Heinrich MC, van de Rijn M. The novel marker, DOG1, is expressed ubiquitously in gastrointestinal stromal tumors irrespective of KIT or PDGFRA mutation status. Am J Pathol. 2004; 165:107–13. 10.1016/S0002-9440(10)63279-8. 15215166 PMC1618538

[9] Referenced with permission from the NCCN Clinical Practice Guidelines in Oncology (NCCN Guidelines®) for Gastrointestinal Stromal Tumors 1.2025. 2025 © National Comprehensive Cancer Network, Inc. 2025. All rights reserved. Accessed [1/11/2026]. To view the most recent and complete version of the guideline, go online to https://www.nccn.org/.

[10] Pasini B, McWhinney SR, Bei T, Matyakhina L, Stergiopoulos S, Muchow M, Boikos SA, Ferrando B, Pacak K, Assie G, Baudin E, Chompret A, Ellison JW, et al. Clinical and molecular genetics of patients with the Carney-Stratakis syndrome and germline mutations of the genes coding for the succinate dehydrogenase subunits SDHB, SDHC, and SDHD. Eur J Hum Genet. 2008; 16:79–88. 10.1038/sj.ejhg.5201904. 17667967

[11] Matyakhina L, Bei TA, McWhinney SR, Pasini B, Cameron S, Gunawan B, Stergiopoulos SG, Boikos S, Muchow M, Dutra A, Pak E, Campo E, Cid MC, et al. Genetics of carney triad: recurrent losses at chromosome 1 but lack of germline mutations in genes associated with paragangliomas and gastrointestinal stromal tumors. J Clin Endocrinol Metab. 2007; 92:2938–43. 10.1210/jc.2007-0797. 17535989

[12] von Mehren M, Kane JM, Riedel RF, Sicklick JK, Pollack SM, Agulnik M, Bui MM, Carr-Ascher J, Choy E, Connelly M, Dry S, Ganjoo KN, Gonzalez RJ, et al. NCCN Guidelines® Insights: Gastrointestinal Stromal Tumors, Version 2.2022. J Natl Compr Canc Netw. 2022; 20:1204–14. 10.6004/jnccn.2022.0058. 36351335 PMC10245542

[13] Debiec-Rychter M, Sciot R, Le Cesne A, Schlemmer M, Hohenberger P, van Oosterom AT, Blay JY, Leyvraz S, Stul M, Casali PG, Zalcberg J, Verweij J, Van Glabbeke M, et al, and EORTC Soft Tissue and Bone Sarcoma Group, and Italian Sarcoma Group, and Australasian GastroIntestinal Trials Group. KIT mutations and dose selection for imatinib in patients with advanced gastrointestinal stromal tumours. Eur J Cancer. 2006; 42:1093–103. 10.1016/j.ejca.2006.01.030. 16624552

[14] Gastrointestinal Stromal Tumor Meta-Analysis Group (MetaGIST). Comparison of two doses of imatinib for the treatment of unresectable or metastatic gastrointestinal stromal tumors: a meta-analysis of 1,640 patients. J Clin Oncol. 2010; 28:1247–53. 10.1200/JCO.2009.24.2099. 20124181 PMC2834472

[15] Heinrich MC, Owzar K, Corless CL, Hollis D, Borden EC, Fletcher CD, Ryan CW, von Mehren M, Blanke CD, Rankin C, Benjamin RS, Bramwell VH, Demetri GD, et al. Correlation of kinase genotype and clinical outcome in the North American Intergroup Phase III Trial of imatinib mesylate for treatment of advanced gastrointestinal stromal tumor: CALGB 150105 Study by Cancer and Leukemia Group B and Southwest Oncology Group. J Clin Oncol. 2008; 26:5360–67. 10.1200/JCO.2008.17.4284. 18955451 PMC2651078

[16] Wozniak A, Rutkowski P, Piskorz A, Ciwoniuk M, Osuch C, Bylina E, Sygut J, Chosia M, Rys J, Urbanczyk K, Kruszewski W, Sowa P, Siedlecki J, et al, and Polish Clinical GIST Registry. Prognostic value of KIT/PDGFRA mutations in gastrointestinal stromal tumours (GIST): Polish Clinical GIST Registry experience. Ann Oncol. 2012; 23:353–60. 10.1093/annonc/mdr127. 21527588

[17] Panizza E, Ercolino T, Mori L, Rapizzi E, Castellano M, Opocher G, Ferrero I, Neumann HP, Mannelli M, Goffrini P. Yeast model for evaluating the pathogenic significance of SDHB, SDHC and SDHD mutations in PHEO-PGL syndrome. Hum Mol Genet. 2013; 22:804–15. 10.1093/hmg/dds487. 23175444

[18] Gill AJ, Chou A, Vilain R, Clarkson A, Lui M, Jin R, Tobias V, Samra J, Goldstein D, Smith C, Sioson L, Parker N, Smith RC, et al. Immunohistochemistry for SDHB divides gastrointestinal stromal tumors (GISTs) into 2 distinct types. Am J Surg Pathol. 2010; 34:636–44. 10.1097/PAS.0b013e3181d6150d. 20305538

[19] Boikos SA, Stratakis CA. The genetic landscape of gastrointestinal stromal tumor lacking *KIT* and *PDGFRA* mutations. Endocrine. 2014; 47:401–8. 10.1007/s12020-014-0346-3. 25027296 PMC4729312

[20] Miettinen M, Lasota J. Succinate dehydrogenase deficient gastrointestinal stromal tumors (GISTs) - a review. Int J Biochem Cell Biol. 2014; 53:514–19. 10.1016/j.biocel.2014.05.033. 24886695 PMC4112081

[21] Casali PG, Blay JY, Abecassis N, Bajpai J, Bauer S, Biagini R, Bielack S, Bonvalot S, Boukovinas I, Bovee JVM, Boye K, Brodowicz T, Buonadonna A, et al, and ESMO Guidelines Committee, EURACAN and GENTURIS. Gastrointestinal stromal tumours: ESMO-EURACAN-GENTURIS Clinical Practice Guidelines for diagnosis, treatment and follow-up. Ann Oncol. 2022; 33:20–33. 10.1016/j.annonc.2021.09.005. 34560242

[22] Joensuu H, Hohenberger P, Corless CL. Gastrointestinal stromal tumour. Lancet. 2013; 382:973–83. 10.1016/S0140-6736(13)60106-3. 23623056

